# Tuning the electrocaloric enhancement near the morphotropic phase boundary in lead-free ceramics

**DOI:** 10.1038/srep28251

**Published:** 2016-06-17

**Authors:** Florian Le Goupil, Ruth McKinnon, Vladimir Koval, Giuseppe Viola, Steve Dunn, Andrey Berenov, Haixue Yan, Neil McN. Alford

**Affiliations:** 1 Department of Materials, Imperial College London, London, SW7 2AZ, UK; 2 School of Engineering and Materials Science, Queen Mary University of London, 380 Mile End Road, London E1 4NS, UK; 3 Institute of Materials Research, Slovak Academy of Sciences, Watsonova 47, 040 01 Kosice, Slovak Republic

## Abstract

The need for more energy-efficient and environmentally-friendly alternatives in the refrigeration industry to meet global emission targets has driven efforts towards materials with a potential for solid state cooling. Adiabatic depolarisation cooling, based on the electrocaloric effect (ECE), is a significant contender for efficient new solid state refrigeration techniques. Some of the highest ECE performances reported are found in compounds close to the morphotropic phase boundary (MPB). This relationship between performance and the MPB makes the ability to tune the position of the MPB an important challenge in electrocaloric research. Here, we report direct ECE measurements performed on MPB tuned NBT-06BT bulk ceramics with a combination of A-site substitutions. We successfully shift the MPB of these lead-free ceramics closer to room temperature, as required for solid state refrigeration, without loss of the criticality of the system and the associated ECE enhancement.

In polar crystals, net polarisation increases under an external electric field. Under adiabatic conditions, the system compensates this alignment of dipoles with an increase in temperature keeping the entropy of the system constant^1^,^2^. This phenomenon is the electrocaloric effect (ECE)^3^,^4^,^5^,^6^,^7^. Lead-free relaxor ferroelectrics with high dielectric strength^8^ are suitable candidates for solid state refrigeration, due to the extra contribution of their polar nano-regions (PNRs), however, their ECE must be increased. One approach is to operate close to critical points (CP) where energy barriers for switching between different phases are reduced. As more than one polar phase coexist near the CP, the entropy of this region is increased. This leads to ECE enhancement^9^, as reported by Qian *et al*. in Zr-doped BaTiO_3_(BT)^10^. It has been shown that an invariant CP, where the number of polar phases in the composition-temperature-electric field phase diagram is maximised, are found in materials with a MPB, as observed in (1 − x)Pb(Mg_1/3_Nb_2/3_)O_3_-xPbTiO_3_ (PMN-xPT) with x ~ 0.30^11^,^12^. The ECE responsivity (ΔT_max_/ΔE) can be used to compare the ECE performances in different materials. It must be taken with caution as it has been shown to strongly depend on both the temperature and the applied field. Rozic *et al*. reported that the ECE responsivity versus applied field reaches a maximum near the critical point (T_CP_/E_CP_) and then slowly decreases with applied field for E > E_CP_^13^. For compositions near the MPB, such as PMN-30PT, the critical ECE responsivity reaches values >0.40 × 10^−6^ K.m/V. However, it decreases <0.30 × 10^−6^ K.m/V for compositions away from the MPB, such as PMN^13^. The value of ECE responsivity at higher electric field is also of interest due to the need for high values of ECE for practical applications. When a higher electric field is applied, corresponding to approximately 2 to 3 times the critical field, the ECE responsivity near the MPB composition of PMN-PT is >0.30 × 10^−6^ K.m/V, while it decreases down to 0.15-0.20 × 10^−6^ K.m/V away from the MPB (PMN)^13^,^14^. In this work, we define an “enhanced” ECE as having a high-field (2–3 × E_CP_) ECE responsivity >0.30 × 10^−6^ K.m/V and a critical (E ~ E_CP_) ECE responsivity >0.40 × 10^−6^ K.m/V. For each composition and electric field, the ECE responsivity is calculated at the temperature of the maximum ECE.

(Na_0.5_Ba_0.5_)TiO_3_ (NBT) forms solid solutions with numerous ferroelectric materials, such as BT or KNbO_3_ (KN) and several MPB have been reported^15^,^16^. The NBT-BT phase diagram shows a MPB near 6%-BT, where rhombohedral, tetragonal and cubic phases co-exist^15^. NBT-BT has the highest electromechanical properties, including polarisation and piezoelectric coefficient near this composition^17^ with NBT-06BT being considered for electrocaloric cooling^18^. However, the temperature of depolarisation (Td) of NBT-06BT or onset of the ECE occurs above 100 °C, too high for household refrigeration applications. Hence, solutions are required to shift the ECE towards room temperature.

Here, we perform direct ECE measurements on NBT-06BT bulk ceramics up to 50 kV/cm while doping the A-site with Ca and Li. Reports^19^,^20^,^21^ show that an increase of Ca or Li contents shift Td to lower temperatures. In this work, we introduce 5% Ca and a range of different concentrations of Li to shift the MPB. This was expected to enhance the ECE and move it closer to room temperature as required for solid state refrigeration. Our material (1−y/100)(0.94Na_0.5−x/100_Li_x/100_Bi_0.5_TiO_3_-0.06BaTiO_3_)-y/100CaTiO_3_ is denoted as (x, y)(Li, Ca)-NBT-06BT.

## Results

**Figure 1:**
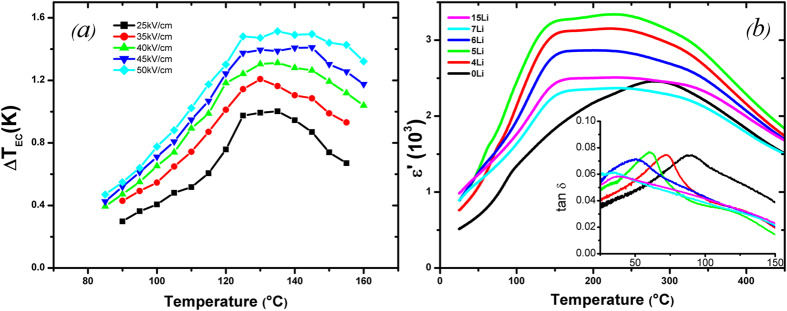
(**a**) ECE versus temperature measured under different values of applied electric field for NBT-06BT. (**b**) Real part of the dielectric permittivity and loss tangent (inset) measured as a function of temperature after a 60 kV/cm-poling on (x, 5)(Li, Ca)-NBT-06BT at 1 kHz for different concentrations of Li.

Figure1(a) shows the ECE/temperature for NBT-06BT under different applied electric fields. A ΔT_max_ of 1.5 K was measured at 135 °C under 50 kV/cm, while ΔT_max_ of 1 K was found under 25 kV/cm. These values are high when compared with other reported bulk ceramics due to the proximity to the MPB of the composition. The few values of electric field studied in this work did not allow for an accurate estimation of the critical electric field. Furthermore, the P-E data seemed to indicate that the critical field was below the lowest value of electric field studied here. Consequently, it was assumed that 25 kV/cm was the value which was the closest to the critical electric field and the critical ECE responsivity was calculated at 25 kV/cm while the high-field ECE responsivity (~2 × E_CP_) was calculated at 50 kV/cm. As expected, the critical ECE responsivity was found to be 0.40 × 10^−6^ K.m/V for NBT-06BT while the high-field ECE responsivity was 0.30 × 10^−6^ K.m/V, which suggests that a similar ECE enhancement is observed near the MPB in NBT-BT as in PMN-PT^14^. Our results also show that the field dependence of the position of ΔT_max_ remains relatively constant at ~135 °C from 25 kV/cm to 50 kV/cm.

Figure 1(b) shows the real part of dielectric permittivity measured for Li-doped samples, x = 0 to 15, after poling at 60 kVcm. The introduction of lithium increases the permittivity, with the maximum values associated with the 4%-Li and 5-%Li samples. Further increasing the Li-content decreased the permittivity to values close to that of the Li-free sample. An anomaly is observed at low temperatures (~90 °C) on the Li-free poled data which is more obvious in the dielectric loss data (insert). This corresponds to the temperature of depolarisation (Td) marking the transition from the long-range ferroelectric regions to short-range PNRs. Figure 1(b) shows that Td shifts to lower temperatures with increasing Li-content. It reaches ~30 °C for 7%-Li and 15%-Li ceramics. The polarisation/temperature data for a variety of Li-contents is shown in supplementary information. Td can be obtained from these data and values of Td were found to be systematically higher than those obtained from dielectric loss data. These discrepancies are due to the difficulty of identifying inflection points in broad and diffuse permittivity data^22^. Encouragingly the trend of decreasing Td with increasing Li-content was confirmed by polarisation data. Td was found be: 103–108 °C, 95–100 °C and 75–85 °C, for 4%-Li, 5%-Li and 6%-Li ceramics, respectively.

**Figure 2:**
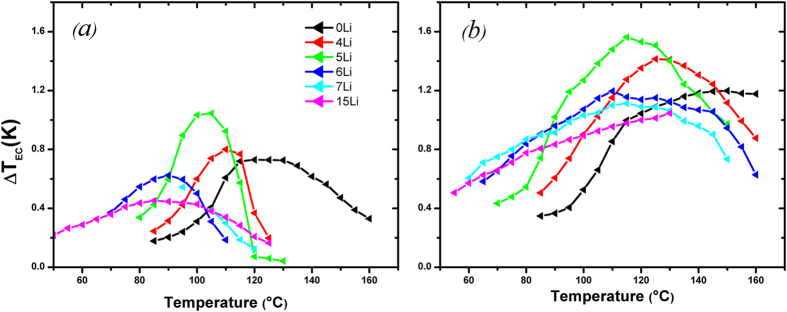
ECE versus temperature measured for (x, 5)(Li, Ca)-NBT-06BT ceramics for different concentrations of lithium under (**a**) 25 kV/cm and (**b**) 50 kV/cm.

Figure 2 shows the ECE/temperature for Li-doped ceramics under (a) 25 kV/cm and (b) 50 kV/cm. ΔT_max_ = 1.2 K is measured at 150 °C under 50 kV/cm for (0, 5)(Li, Ca)-NBT-06BT, which corresponds to a high-field ECE responsivity of 0.24 × 10^−6^ K.m/V and marks a 20% decrease *cf*. the Ca-free ceramic. Similarly, the critical ECE responsivity decreases down to 0.29 × 10^−6^ K.m/V. It is also clear that the field dependence of ΔT_max_ is higher than for NBT-06BT. ΔT_max_ = 0.73 K at 120 °C and 25 kV/cm, shifts to higher temperatures under stronger fields as observed in relaxor ferroelectrics^14^,^23^. This shift is due to the contribution to the ECE of the nucleation and alignment of PNRs at higher electric fields^24^. The shape of the peak suggests a shift to the relaxor side of the phase diagram^23^. This has detrimental effects on the ECE performance: ΔT_max_ decreased, due to a shift away from the MPB and although Td shifts to lower temperatures, the position of the ECE peak at higher fields shifts to higher temperatures due to the relaxor- behaviour induced by Ca-doping. Figure 2(a) shows that the introduction of Li in the NBT-based material shifted the Td and ECE peak to lower temperatures. The maximum ECE under a 25 kV/cm-applied field shifts from 120 °C (Li-free) to 85 °C ((15, 5)(Li, Ca)-NBT-06BT). The 25 kV/cm data show a change of the ECE peak shape as the Li content increases. With 4% and 5% of lithium the peak is sharper than for the Li-free samples. A further increase in Li-content, 6%, 7% and 15%, gives a broadening of the ECE peak. The maximum ECE enhancement was observed for the 4%-Li and 5%-Li samples. As shown in the supplementary information, the critical ECE responsivity of the 5%-Li sample was found to be even higher (0.42 × 10^−6^ K.m/V) than that of the MPB composition. While ΔT_max_ rises from 0.73 K for Li-free samples to 0.80 K for 4%-Li and 1.05 K for 5%-Li the broadening of the ECE peak associated with 6%-Li, 7%-Li and 15%-Li has a detrimental effect on the ECE. In these cases it decreases to 0.62 K, 0.54 K and 0.45 K.

The trend for ΔT_max_ versus Li concentration for the 50 kV/cm data is shown in Fig. 2(b). Similar trends are exhibited in this high field range with ΔT_max_ rising from 1.20 K for the Li-free material to 1.41 K for 4%-Li and 1.56 K for 5%-Li, and decreasing for the Li-rich ceramics (>6%), with a broadening of the ECE peak. The corresponding high-field ECE responsivity for 5%-Li is 0.31 × 10^−6^ K.m/V, which suggests that the ECE enhancement observed near the MPB composition in NBT-06BT can be recovered at lower temperatures when a critical concentration of lithium is introduced. The broadening of the ECE response in Li-rich ceramics increases the operational range (~80 °C) of the cooling cycle for a single composition, which could eliminate the need for multi-composition systems. However, there is an associated impact on ΔT_max._. When x increased from 5% to 6%, ΔT_max_ (50 kV/cm) drops by ~25%. Furthermore, although the ECE peak at 25 kV/cm shifts to lower temperatures by ~20 °C we show that ΔT_max_ (50 kV/cm) for 6%-Li occurs only 5 °C below that of 5%-Li. This emphasises the need to maintain the system near the MPB for the best performances.

**Figure 3:**
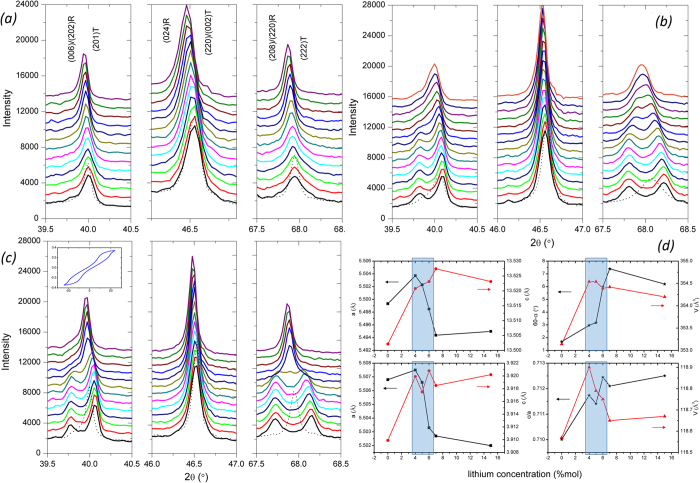
X-ray diffractogram of three relevant peaks measured on (**a**) the unpoled 5%-Li-doped ceramic and the 50 kV/cm-poled (**b**) Li-free ceramics and (**c**) 5%-Li-doped as a function of temperature. The data in black was taken at room temperature, and the temperature was then increased from 40 °C to 150 °C, with 10 °C increments. The black doted data was measured at room temperature after the system cooled back down. (**d**) Lattice parameters, volumes and distortions of the unpoled Li-doped ceramics of the rhombohedral (top) and the tetragonal (bottom) phases at RT for different Li-contents. The highlighted areas mark the compositions with enhanced ECE.

Figure 3(a) shows the X-ray diffractogram measured on the unpoled 5%-Li-doped ceramics versus temperature. The structure is pseudo-cubic over the range of temperature with no obvious peak splitting associated with rhombohedral or tetragonal phases observed. This is consistent with the structure of unpoled NBT-BT ceramics near the MPB being a mixture of polar nanodomains with *R3c* and *P4bm* local symmetry in an overall cubic matrix^25^. This mix of local symmetries is confirmed by our Rietveld refinement results as shown in Table 1. There are no obvious structural changes around Td (~100 °C). However, significant differences are observed in the poled samples, as shown in Fig. 3(b,c). The application of a strong electric field induces the irreversible formation of a long-range order FE phase, with a *R3c* or *P4mm* symmetry, depending on the composition^25^,^26^. Splitting of the (006)/(202)R (~40°) and the (208)/(220)R (~68°) peaks in the hexagonal representation is observed while the (024)R peak (~46°) remains unchanged, which suggests a field-induced rhombohedral phase (*R3c*). It can be seen that the structure of the 5%-Li-doped ceramic sharply reverts back to pseudo-cubic between 90 °C and 110 °C, corresponding to the position of Td. The rhombohedral polar phase can be induced above Td when an electric field ~50 kV/cm is applied as evidenced by the antiferroelectric-like hysteresis loops, shown in the inset of Fig. 3(c). The structure remains pseudo-cubic upon cooling to room temperature when no electric field is applied. Figure 3(b) shows that the structural changes observed near Td (~120 °C) for Li-free samples are more diffuse than those observed for the 5%-Li doped ceramic. The system reverts back to pseudo-cubic from 100 °C to 160 °C. This more diffuse process agrees with the shape of the ECE peaks in Fig. 2(a).

Hiruma *et al*. reported that the position of the MPB in (1−x)NBT-xABO3 materials depends on the tolerance factor of the end-member perovskite^27^. The A-site substitution of a small ion such as Ca^2+^ will require a larger amount of Ba^2+^ to induce the MPB. Using the formula proposed by Hiruma *et al*.^27^, we estimate that the introduction of 5% of Ca moves the MPB to 8%-Ba, instead of the 6% originally present. This explains the detrimental effect we find in the ECE properties. Due to its small ionic radius (1.25Å), Li^+^ is expected to decrease the tolerance factor of the end member and move the MPB towards the Ba-rich side of the phase diagram. As, we maintain the barium content in our study, we would effectively drive the system away from MPB with increasing Li. However, it has been reported for NBT-KBT that the introduction of small amounts of Li increases lattice parameters and distortions of both the rhombohedral and tetragonal phases near the MPB. The small ionic radius of Li^+^ initially makes it difficult to substitute Na^+^ in the A-site of NBT^28^. This increase of lattice distortion affects ferroelectric properties such as the position of Td, while maintaining the position of the MPB. Our structural analysis indicates that Li substitution has a similar effect on NBT-BT. Figure 3(d) shows an increase of the lattice parameters, volumes and distortions after the introduction of up to 5–6% of Li. At higher Li-contents, the fundamental lattice parameters start decreasing and the ECE enhancement is lost. The move away from the MPB at high Li-contents is also evidenced by the significant change of the R/T phase ratio as shown in Table 1.

Our observations show that the introduction of Ca in the NBT-06BT drives the system away from the MPB inducing a relaxor-type phase with a diffuse phase transition. The introduction of Li brings the system back to MPB by reducing the amount of Ba necessary to induce MPB. Due to the proximity of the MPB the field-induced rhombohedral phase in the 5-%Li doped ceramic shows a higher polarisation and sharper depolarisation process at Td, leading to an enhanced ECE. This enhancement is maintained tens of degrees above Td due to the high-entropy of the zero-field weakly polar phase found at higher temperatures.

**Figure 4:**
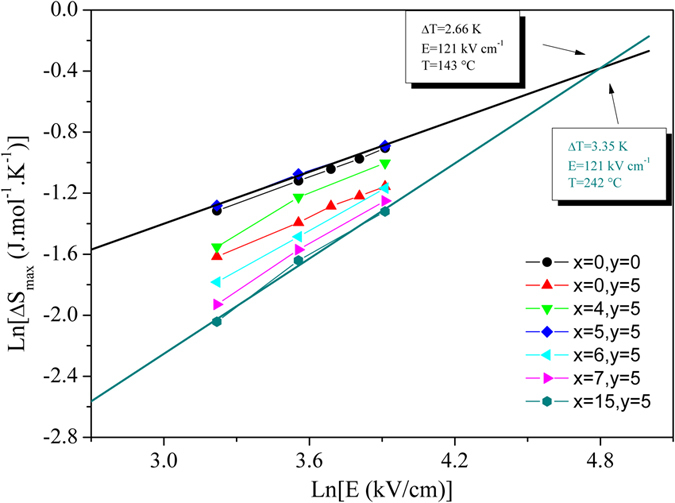
Maximum electrocaloric entropy change (ΔS_max_) as a function of the applied electric field in a logarithmic scale for all the studied compositions.

Figure 4 shows the maximum electrocaloric entropy change (ΔS_max_) versus applied electric field in a logarithmic scale for all studied compositions. ΔS_max_ increases linearly with E in a logarithmic scale, which makes the comparison between the different compositions easier. We show that both the absolute values and field-dependence of the 5%-Li-doped ceramic are similar to that of undoped MPB composition NBT-06BT. This is further confirmation that both Td and the MPB were shifted towards room temperature in the 5%-Li-doped ceramic. Figure 4 shows that the field-dependence of ΔS_max_ is more pronounced in the Li-rich compositions (x > 6%) due to strong relaxor properties. It also shows that if a high enough field is applied (~120 kV/cm), all compositions give similar values of ΔS_max_. However, the strong field-dependence of ECE peak position in the Li-rich compositions means that the maximum ΔS_max_ would be obtained 100 °C higher in the 15%Li-doped system compared to the 5%Li-doped one. This last observation highlights that, although the broad peak induced by the relaxor properties is attractive for cooling applications, the ECE reduction and the significant shift of ΔT_max_ to high temperatures make this compromise unacceptable. The most efficient way to broaden a sharp ECE peak is to use a multilayer-capacitor (MLC) structure. The high fields that can be applied due to the thin active layers (~10 μm) not only increase the absolute values of the ECE, but also broaden the ECE peak. Ye *et al*. reported a high ECE (ΔT_max_ ~ 7 K) spreading over a temperature range of 40 °C^29^. Due to the use of a MLC structure the ECE was obtained by applying a voltage of ~240 V. Hence, a MLC of a composition where the MPB properties were shifted towards room temperature, would combine all the requirements for commercial electrocaloric cooling: an enhanced ECE spread over a broad temperature range in the vicinity of room temperature obtained in a highly energy efficient process.

In conclusion, our dielectric, electrocaloric and structural studies have shown that the combination of several A-site dopings in NBT-06BT can bring the MPB, and the accompanied polarisation and ECE enhancement, closer to room temperature. The A-site doping of both calcium and lithium allowed to conserve, and even increase, the high ECE and ECE responsivity of NBT-06BT, while shifting the temperature of operation closer to room temperature. Many other types of A-site doping, including large ions such as K^+^, still need to be explored for a complete shift to room temperature, but these results are very promising. A good alternate method could be the introduction of smaller ions such Al^3+^ in the B-site, which have also been shown to induce MPB at lower temperatures^27^. Hence, a NBT-KN-BaAl_0.5_Nb_0.5_O_3_ ternary system could be the key to highly-efficient solid-state refrigeration near room temperature. The ability to tune the position of the MPB demonstrated here has major implications for the future of the electrocaloric research. It will allow the use of the materials with the highest ECE performances without any loss in efficiency at any required temperature of operation, whether it is near 25 °C for household refrigeration or 200 °C for on-chip cooling in advanced electronics.

## Methods

Ceramic powders with composition (1-y)[0.94(Bi_0.5_Na_(0.5−*x*)_Li_*x*_)TiO_3_-0.06BaTiO_3_]-*y*CaTiO_3_ (*x* = 0, 0.04, 0.05, 0.06, 0.07, 0.15 and *y* = 0, 0.05) were prepared by solid-state reaction from high-purity oxides and carbonates: Bi_2_O_3_ (99.9% Sigma-Aldrich), TiO_2_ (99.8% Sigma-Aldrich), Na_2_CO_3_ (99.5% Alfa Aesar), BaCO_3_ (99.8% Alfa Aesar), CaCO_3_ (99.5% Alfa Aesar) and Li_2_CO_3_ (99.0% Alfa Aesar). The dried powder mixtures were then calcinated at 800–1000 °C for 4 h. Compact pellets were sintered in air at 1150–1200 °C for 2 h.

X-ray diffraction (XRD) was performed on crushed ceramics using a Siemens D5000 diffractometer (Siemens AG, Karlsruhe, Germany) operating at 40 kV and 30 mA with Cu Kα radiation. Data from the X-ray diffractometer of powders were analyzed by the Rietveld method^30^ using the FullProf program^31^.

For the electrical characterisation of the ceramics, silver paste (Gwent Electronic Material Ltd., C2011004D5, Pontypool, UK) was fired at 600 °C for 10 mins onto the ceramic surface to form electrodes. The dielectric permittivity and loss were measured with an LCR meter (Agilent, 4284A, Hyogo, Japan) between 25 °C and 600 °C for frequencies between 1 kHz and 500 kHz. Current-polarisation-electric field (I-P-E) loops were measured with a hysteresis tester (NPL, Teddington, UK) between 25 °C and 150 °C. The direct ECE measurements were performed with a modified-Differential Scanning Calorimeter (Netzsch DSC 200 F3), as described elsewhere^5^. The composition dependence of the specific heat capacity, which is given in the supplementary information, was found to be very small with values increasing with temperature from 440 to 560 J.kg^−1^.K^−1^.

## Additional Information

**How to cite this article**: Le Goupil, F. *et al*. Tuning the electrocaloric enhancement near the morphotropic phase boundary in lead-free ceramics. *Sci. Rep*. **6**, 28251; doi: 10.1038/srep28251 (2016).

## Supplementary Material

Supplementary Information

## Figures and Tables

**Table 1:** Repartition (wt%) of rhombohedral (*R3c*) and tetragonal (*P4bm*) phases versus lithium content.

	MPB	0	4	5	6	7	15
*R3c*	45	46	42	41	38	33	31
*P4bm*	55	54	58	59	62	67	69
