# Supplementary material for: Tuning the electrocaloric enhancement near the morphotropic phase boundary in lead-free ceramics
Source: Sci Rep. 2016 Jun 17;6:28251. doi: 10.1038/srep28251 (PMC4911558; doi:10.1038/srep28251)
Supplement: Supplementary Information [file srep28251-s1.doc]

Tuning the electrocaloric enhancement near the morphotropic phase boundary in lead-free ceramics.

Florian Le Goupil^1,*^, Ruth McKinnon^2^, Vladimir Koval^3^, Giuseppe Viola^2^, Steve Dunn^2,*^, Andrey Berenov^1^, Haixue Yan^2^ and Neil McN. Alford^1^.

^1^ Department of Materials, Imperial College London, London, SW7 2AZ, UK

^2^ School of Engineering and Materials Science, Queen Mary University of London, 380 Mile End Road, London E1 4NS, UK

^3^ Institute of Materials Research, Slovak Academy of Sciences, Watsonova 47, 040 01 Kosice, Slovak Republic

Correspondence should be addressed to F.L. (email:f.le-goupil09@imperial.ac.uk).

Supplementary Information


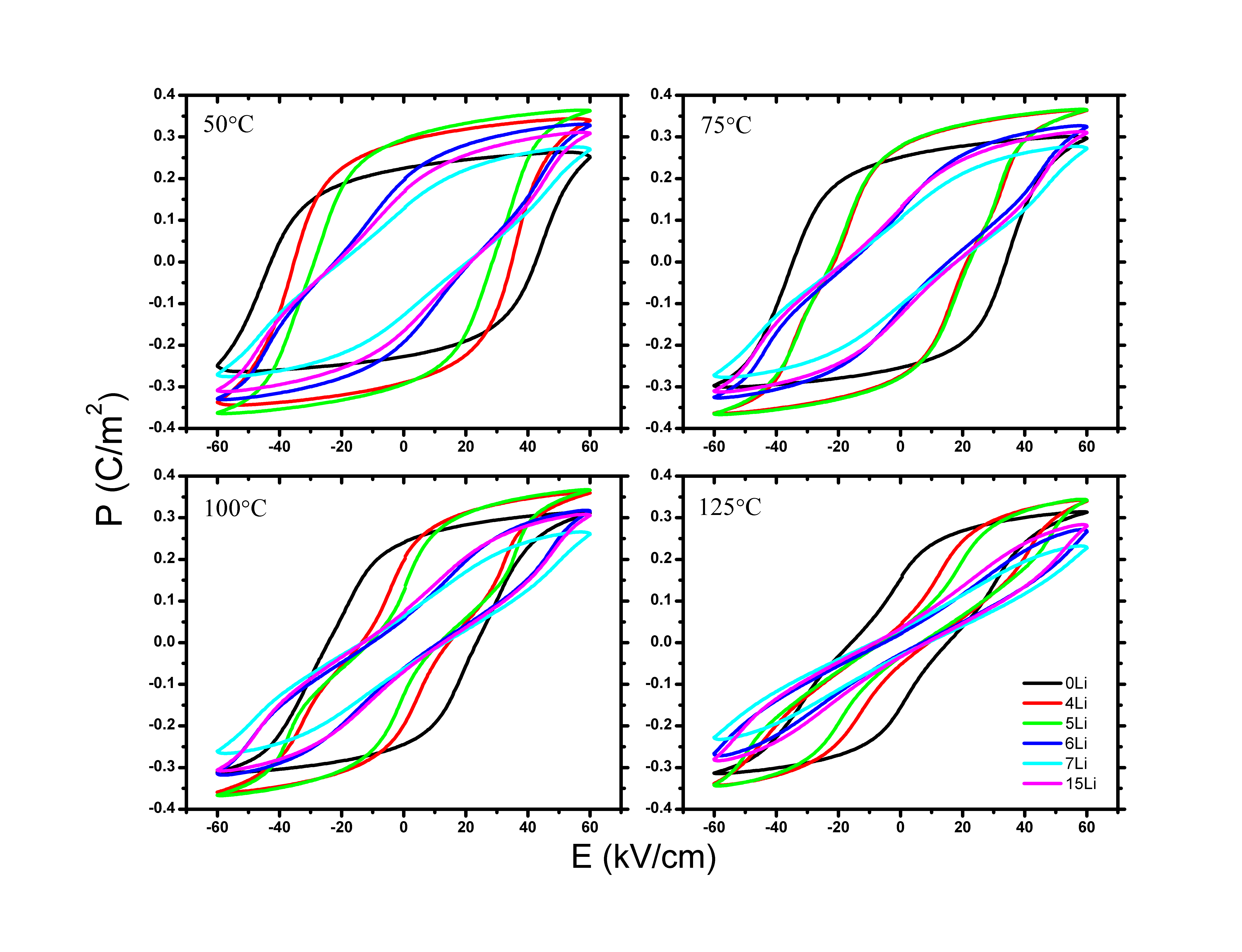


**Figure 1. Polarisation versus electric field measured for (x,5)(Li,Ca)-NBT-06BT ceramics for different concentrations of lithium doping at four different temperatures.**


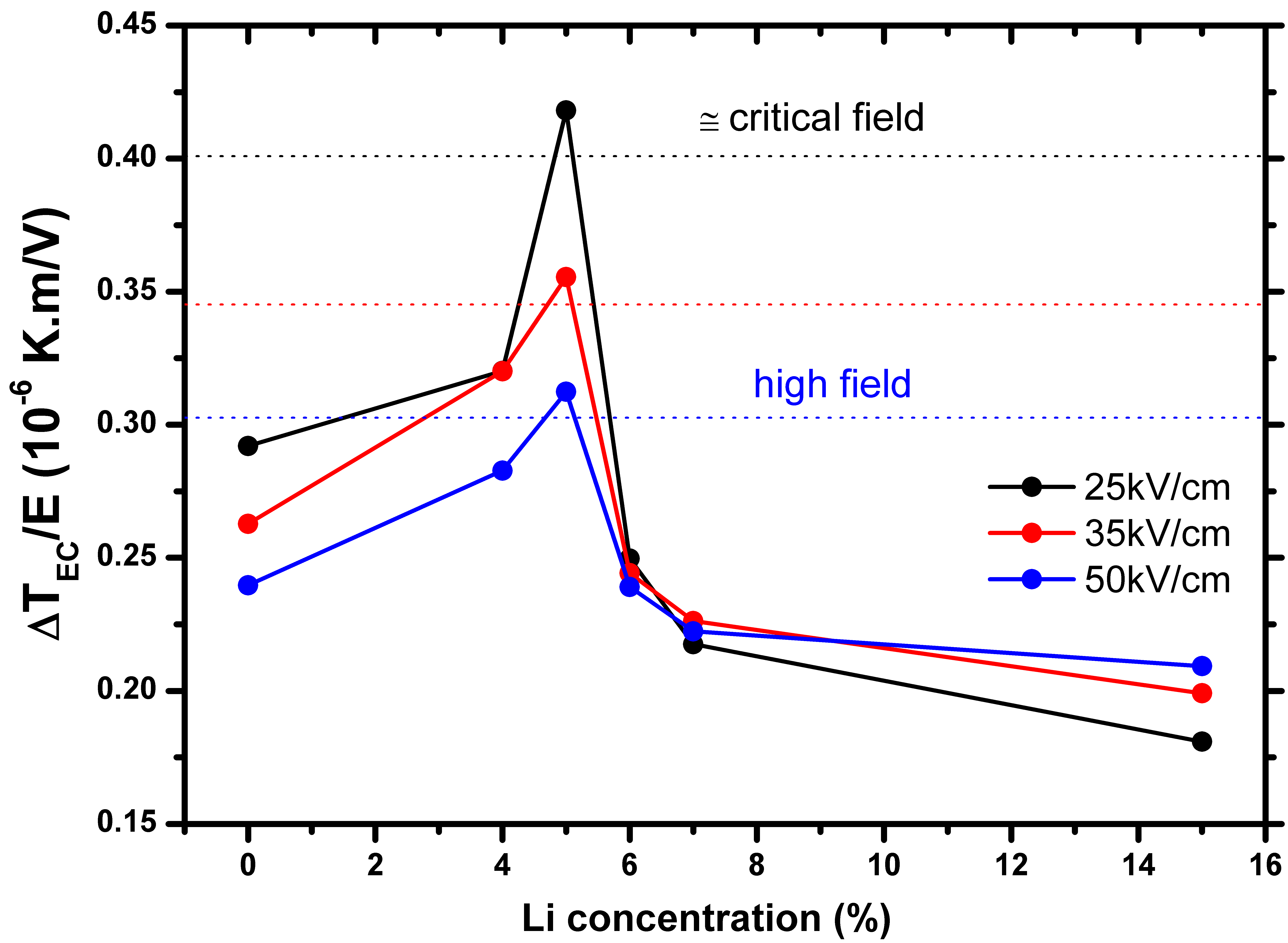


**Figure 2. Electrocaloric responsivity as a function of lithium concentration measured at the temperature of maximum ECE for several values of applied electric field, including the near critical field. For each electric field the dotted line marks the ECE responsivity obtained for the MPB composition NBT-06BT.**


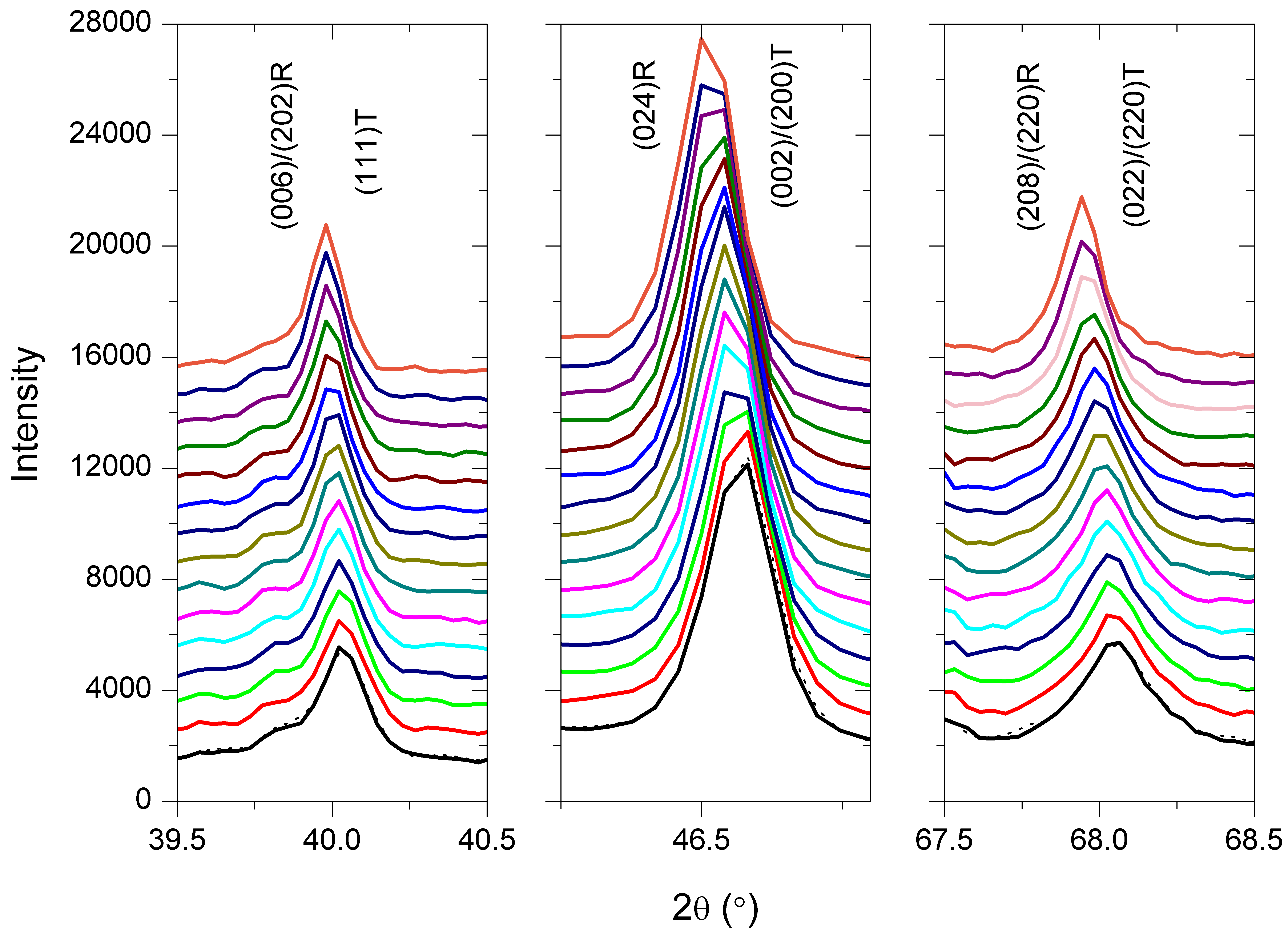


**Figure 3. X-ray diffractogram of three relevant peaks measured on the Li-free ceramic, as a function of temperature. The data in black corresponds to the room temperature, and the temperature is then increased from 40°C to 170°C, with 10°C increments. The black doted data corresponds to the room temperature measurement performed after the system cooled back down.**


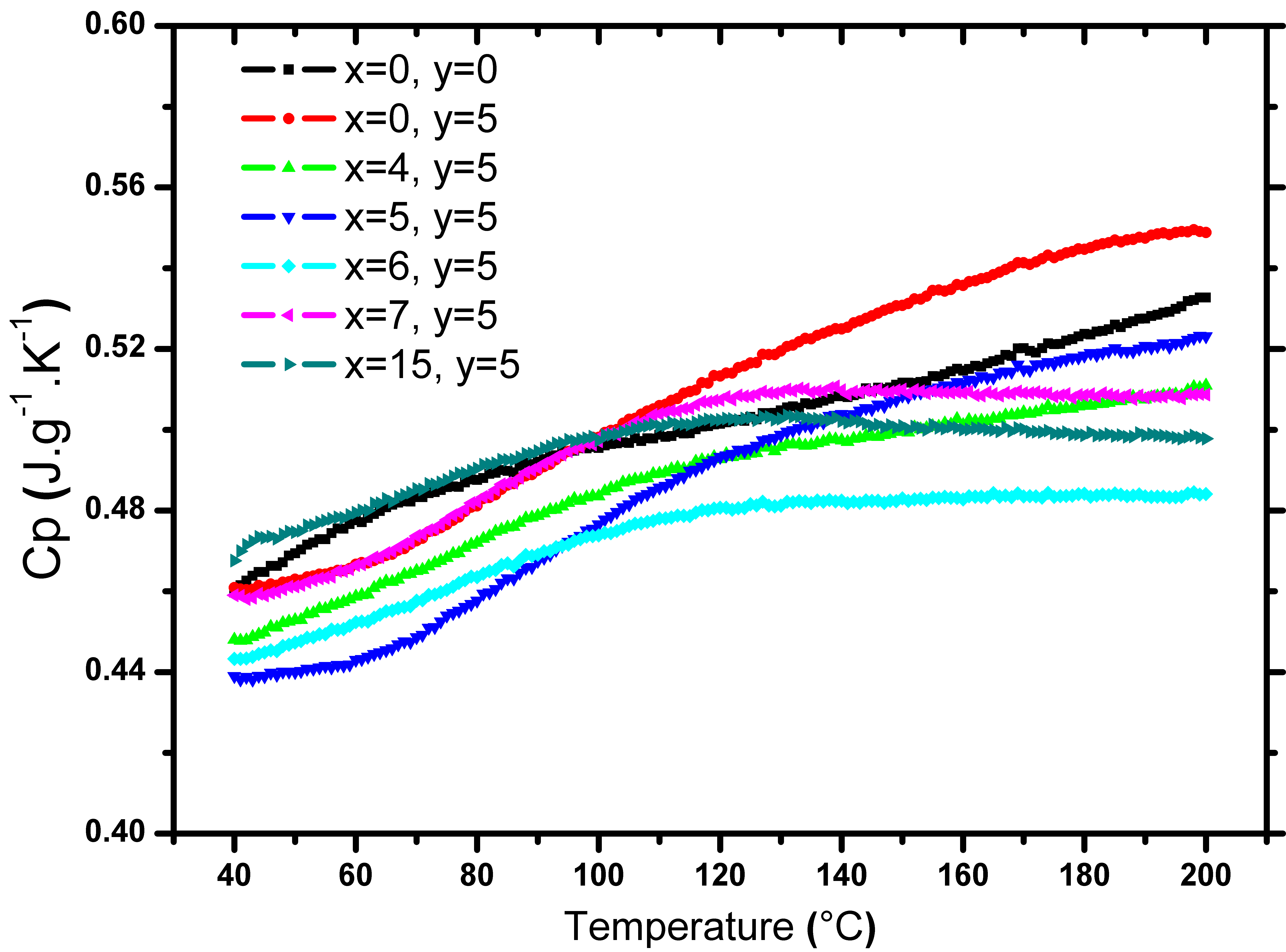


**Figure 4. Specific heat capacity as a function of temperature measured for all the studied compositions.**
